# Clinical and multiple gene expression variables in survival analysis of breast cancer: Analysis with the hypertabastic survival model

**DOI:** 10.1186/1755-8794-5-63

**Published:** 2012-12-14

**Authors:** Mohammad A Tabatabai, Wayne M Eby, Nadim Nimeh, Hong Li, Karan P Singh

**Affiliations:** 1 Department of Mathematical Sciences, Cameron University, Lawton, OK, 73505, USA; 2 Cancer Centers of Southwest Oklahoma, Lawton, OK, 73505, USA; 3 Department of Medicine, University of Alabama at Birmingham, Birmingham, AL, 35295, USA

**Keywords:** Hypertabastic survival models, Gene expression variables, Breast cancer biomarkers, Seventy gene signature, ErbB2 overexpression, Fibroblast core serum response

## Abstract

**Background:**

We explore the benefits of applying a new proportional hazard model to analyze survival of breast cancer patients. As a parametric model, the hypertabastic survival model offers a closer fit to experimental data than Cox regression, and furthermore provides explicit survival and hazard functions which can be used as additional tools in the survival analysis. In addition, one of our main concerns is utilization of multiple gene expression variables. Our analysis treats the important issue of interaction of different gene signatures in the survival analysis.

**Methods:**

The hypertabastic proportional hazards model was applied in survival analysis of breast cancer patients. This model was compared, using statistical measures of goodness of fit, with models based on the semi-parametric Cox proportional hazards model and the parametric log-logistic and Weibull models. The explicit functions for hazard and survival were then used to analyze the dynamic behavior of hazard and survival functions.

**Results:**

The hypertabastic model provided the best fit among all the models considered. Use of multiple gene expression variables also provided a considerable improvement in the goodness of fit of the model, as compared to use of only one. By utilizing the explicit survival and hazard functions provided by the model, we were able to determine the magnitude of the maximum rate of increase in hazard, and the maximum rate of decrease in survival, as well as the times when these occurred. We explore the influence of each gene expression variable on these extrema. Furthermore, in the cases of continuous gene expression variables, represented by a measure of correlation, we were able to investigate the dynamics with respect to changes in gene expression.

**Conclusions:**

We observed that use of three different gene signatures in the model provided a greater combined effect and allowed us to assess the relative importance of each in determination of outcome in this data set. These results point to the potential to combine gene signatures to a greater effect in cases where each gene signature represents some distinct aspect of the cancer biology. Furthermore we conclude that the hypertabastic survival models can be an effective survival analysis tool for breast cancer patients.

## Background

A number of important papers have appeared in recent years using gene expression as a predictor of outcome in cancer patients, and it has become clear this genomic information will greatly improve prognostic capabilities. In the statistical survival analysis, these papers have utilized the semi-parametric Cox proportional hazard model and the Kaplan-Meiers estimator for the survival and hazard curves. One purpose of this paper is to show the advantages that can be gained by utilizing a parametric model, which allows use of explicitly defined, continuous hazard and survival functions for tools in analysis. Parametric models in general have a higher accuracy, and the recently introduced hypertabastic model [1] is shown to provide the best fit for the data set under consideration, among the other competing parametric models of Weibull and log-logistic. Although there may sometimes be a concern in using a parametric model rather than the semi-parametric Cox model in cases where the distribution of the data is unknown, these models have greater accuracy and provide more detailed information when they are applicable. The hypertabastic model has been shown to be robust with respect to departure of the data from the distribution [1,2], making it an appropriate model to use in describing a wide variety of survival data. This model has also been shown to provide a good fit to breast cancer survival data in a recent paper [3]. Using the explicit hazard and survival functions provided by this model we demonstrate some of the potential for analysis of temporal dynamics of the progression of hazard and decrease in survival. We are able to use the survival function to explicitly compute probability of survival to a given time, and this prediction takes into account an individual patient’s profile with respect to any significant variables included in the model.

Breast cancer patients with similar clinical profiles may experience widely differing outcomes and different responses to therapy, and means for more accuracy in prognosis will fill an important need. The development of variables with more prognostic power was a primary goal in the development of gene expression signatures for breast cancer outcome. Early papers utilizing gene expression to predict the progression of breast cancer determined several distinct categories [4], which have become linked to molecular subtype. The different molecular subtypes had different prognoses, with basal-like and ErbB2+ tumors experiencing more invasive tumors and increased risk of recurrence, while the luminal subtype are characterized by less invasiveness and a better response to treatment. Luminal tumors were later subdivided [4] into Lumina A and Lumina B, with distinct prognosis. The authors [5] used microarrays and statistical methods to determine a list of genes whose expression correlated strongly to a positive outcome for the patients, based on short term distant metastasis. This research established a 70 gene signature which could be used for prognosis of tumors as poor or good outcome. Many other teams of researchers, such as [6,7], have also used similar methods to establish a gene expression signature highly correlated to patient outcome. Based on the older idea [8] that tumors and wounds produce a similar microenvironment which facilitates proliferation and migration of cells and stimulates angiogenesis, the papers of Chang and collaborators [9,10] determined prognostic capabilities of gene expression signatures associated to wound healing.

More recently researchers [11,12] have addressed issues of developing these methods for use together with standard variables for prognosis in clinical cases. In particular, [12] used model selection with Cox regression to determine the best set of predictors from among the standard clinical variables a collection of hundreds of gene signatures. These researchers came to the conclusion that gene expression variables are the most powerful predictors, and most of these gene signatures are comparable to the others in prognostic power. However, addition of clinical variables to the model displayed a small increase in the power of the model. Other researchers [13-15] have also noted that different gene expression signatures carry much of the same information. These researchers do not expect use of several different signatures to yield much improvement in prognosis. However, we note that Chang et al. [10] proposed use of both the seventy gene signature and the wound expression gene signature to a combined effect in prediction of patient risk. Furthermore the work of [16] develops a computational approach for prognosis which uses both gene expression and a means of classification into molecular subtype. The current study investigates the interaction between clinical variables and several gene signatures as predictors for outcome in breast cancer patients. We have found that combining several gene expression variables provides a model that best fits the survival data. Consistent with the results of Chang et al. [10] the model uses the seventy gene signature of [5] together with core serum response, a wound healing signature developed in [9]. In addition one of the gene expression signatures from [4] for classification into molecular subtype is shown to be statistically significant. This particular gene signature for ErbB2+ overexpression also relates to important aspects of the underlying breast cancer tumor biology explored by numerous researchers. The issue of what happens in the interactions of several significant gene expression variables also arises inherently in these considerations.

Clinical trials have begun for gene expression signatures in breast cancer [17,18], and these biomarkers can be expected to soon become available for use in the clinical setting. Furthermore researchers have begun development of a second generation of gene expression signatures, including analysis of signatures from nearby stromal cells [19], immune response [20], and mutations in cancer related pathways [21]. Gene expression profiles have additionally been developed for other aspects of breast cancer therapy response [22], including response to radiotherapy and response to chemotherapy [23-26].

The combined model we form in this paper illustrates how a quantitative prediction of hazard and survival can be formed that incorporates the predictive capabilities of these three gene expression variables. Note that each of these variables has medical significance in breast cancer progression. In our discussion of this model in the Results and discussion section, we explore the role of these variables, how they affect one another in the context of the xmodel, and what information can be gained from variation in the levels of CSR correlation, ErbB2+ correlation, and good or poor seventy gene signature. This analysis and investigation addresses the important issue of how multiple gene expression signatures representing different aspects of the underlying biology can be combined and how they may interact. We have found a partial answer in the context of the given model; however it is far from complete in answering this important question. We claim this is an important issue that should receive further attention and possibly alternative approaches in modeling.

## Methods

Here we present the proportional hazard form of the Hypertabastic model, which will be applied in the survival analysis of the breast cancer patients. One important feature of the hypertabastic survival model is the ability of the hazard function to assume many different shapes, in contrast to the Weibull, lognormal, and log logistic distributions. The hypertabastic distribution function is defined as

$$F\left(t\right)=\{\begin{array}{c} 1-sech\left(\alpha \left[1-t^{\beta }coth\left(t^{\beta }\right)\right]/\beta \right)\\ 0 \end{array}\begin{array}{c} t>0\\ t\leq 0. \end{array}$$

The hypertabastic proportional hazard model has a hazard function of the form

(1) $$h\left(t|x,\theta \right)=h_{0}\left(t\right)g\left(x|\theta \right)$$

where h_0_(t) is the baseline hazard function, given by

$$h_{0}\left(t\right)=\alpha \left[t^{2\beta -1}csch^{2}\left(t^{\beta }\right)-t^{\beta -1}coth\left(t^{\beta }\right)\right]tanh\left[W\left(t\right)\right]$$

and where *W*(*t*) = *α*[1 − *t*^*β*^*coth*(*t*^*β*^)]/*β*, and *α*, *β* > 0. These parameters α and β provide the flexibility of the hazard function to conform to the given data set. See [1] for examples of different distribution shapes associated to different values of these parameters. The function *g*(*x*|*θ*) is given by *g*(*x*|*θ*) = *Exp*[∑_*k* = 1_^*p*^*θ*_*k*_*x*_*k*_, where the x_k_ are covariates and the θ_k_ are the associated parameters. Similarly the hypertabastic survival function *S*(*t*|*x*, *θ*) for the proportional hazards model has the form

(2) $$S\left(t|x,\theta \right)={\left[S_{0}\left(t\right)\right]}^{g\left(x|\theta \right)}$$

where S_0_(t) is the baseline survival function, given by

$$S_{0}\left(t\right)=sech\left\{\alpha \left[1-t^{\beta }coth\left(t^{\beta }\right)\right]/\beta \right\}\text{.}$$

For further detail, see [1,2]. Simulation studies with this model [2] have demonstrated some degree of robustness with respect to variations in the distribution of the data.

This model is applied to the 295 patient study from the Netherlands Cancer Institute which is presented in [27] as a validation set for the seventy gene signature. All of these patients had stage I or II breast cancer but had no previous history of cancer. The study combined both lymph-node positive and lymph-node negative patients. All of these patients had been treated by modified radical mastectomy or breast-conserving surgery. Of the patients with lymph-node positive disease, 120 were treated with adjuvant chemotherapy and/or hormonal-therapy. For more information regarding this study, see [27].

Here we further discuss the different variables that were included as potential covariates in the model. The first class of variables was the clinical variables, including the following: estrogen receptor status (ERS), tumor grade (TG1 and TG2), age (AGE), diameter (DIAM), and lymph node status (LN1 and LN2). The primary gene expression variable we tested was the seventy gene signature (70G) of [5] which selected genes for prediction of early distant metastasis. From the study of the wound healing microenvironment by Chang et al. [9,10], the wound response signature (WRS) and the core serum response correlation (CSR) were included as potential gene expression variables. The core serum response is developed in [9] to represent a canonical expression of fibroblasts activated by serum, and it is a cell-cycle independent set of genes in areas including vascularization, cell motility, and matrix remodeling, common to both the wound healing and tumor microenvironments. Finally, in the area of gene expression for classification of molecular subtype, we considered correlation used for validation in [27] (CVal), and with centroids for normal (CNorm), ErbB2+ (CERBB), Lumina A (CLumA), Lumina B (CLumB), and basal (CBas) from [6].

In implementation of the hypertabastic survival model to this set of data, we considered the clinical, gene expression, and classification variables described above. We applied a standard stepwise forward selection of variables procedure. In addition since some of the variables are highly correlated, we used a procedure that would ensure no two of the variables considered would have a pairwise correlation of 0.5 or higher. The parameters were estimated using a SAS program, and these parameter estimates were double checked using Mathematica. A SAS program for hypertabastic proportional hazard model using log-time is provided in the Additional file 1: Documents.

Once the parameters had been estimated, these values were used in the survival function (2) and hazard function (1). Then Mathematica was utilized to sketch graphs of the hazard and survival functions for the desired cases. Further dynamic analysis of these curves and their derivatives was also made using Mathematica.

## Results and discussion

### Model based on gene expression and clinical variables

In this section we apply the model selection procedure to determine an effective model to represent the survival of the breast cancer patients in the Netherlands study of [27], described briefly above. In selecting from among the hypertabastic, log-logistic, and Weibull proportional hazard models, we compare these models using the −2 log-likelihood score and the Akaike Information Criterion (AIC) [28]. The Akaike Information Criterion is commonly used when selecting among several competing models, with the `smallest value corresponding to the best fit model. See Table 1 where we make a comparison of the three parametric distributions mentioned above. For purposes of comparison, we also include Cox regression. The covariates included in the model include AGE, 70G, CSR, and CERBB.

**Table 1:** Comparison of models

	**−2 Log likelihood**	**AIC**	**−2 Log likelihood without covariates**
Hypertabastic	387.755	399.755	467.952
Weibull	399.000	411.000	474.089
Log Logistic	502.126	514.126	544.930
Cox Regression	764.001	772.001	836.598

In Table 2 we give the estimates a, b for the parameters α and β of the hypertabastic distribution and each of the model variables Age, Seventy gene signature, CSR correlation, and ErbB2+ correlation, together with the standard error, Wald test value and p-value. Among the three gene expression variables included in the model, CSR correlation (CSR) is clearly the most significant with the highest hazard ratio and smallest p-value.

**Table 2:** Parameter estimates and statistical significance for combined model

**Parameter**	**Estimate**	**Standard Dev.**	**Wald test**	**P-value**	**Hazard ratio**
a (model)	0.7247	0.2888	6.298	0.01209	NA
b (model)	0.6205	0.1244	24.873	6.125 10^-7	NA
c (AGE)	−0.07350	0.01480	24.645	6.891 10^-7	0.9291
d (70G)	1.199	0.3872	9.585	0.001962	3.316
e (CSR)	2.661	0.7025	14.343	0.0001524	14.305
f (CERBB)	1.561	0.7285	4.594	0.03208	4.766

Inclusion of the clinical variables improved the goodness of fit of the model for each of the gene signatures considered, consistent with the results of [12]. Although the seventy gene signature gives the best fitting model of all gene expression variables when considered alone, there is a considerable improvement from inclusion of multiple gene expression variables. The combined model features the gene expression variables 70G, representing distant early metastasis, CSR, representing relation to a wound healing microenvironment which promotes cell migration and vascularization, and CERBB, representing ErbB2+/Her2 over-expression and relating to molecular subtype. The individual gene signatures of 70G, CSR, and CERBB yield models with values of AIC of 423.142, 436.056, and 448.248, respectively. However the combined model has a dramatic improvement, to 399.755. Since these signatures represent different aspects of the underlying cancer biology, it is perhaps not surprising the combination of the variables produces a model with a better fit to the data.

In the absence of a combined model, researchers and doctors are already aware of the possibility for several important variables to point toward different conclusions. Our combined model addresses this question of how much weight to assign to each of several significant variables. This model offers a scientific approach to this issue, based on statistical techniques and quantitative analysis. The added advantage of use of a good-fitting parametric model, such as the hypertabastic survival model, is the ability to analyze the temporal dynamics of the hazard and survival functions, as we illustrate in the remainder of this section. Since two of the gene expression variables are continuous, as given by levels of correlation to an established gene expression, we are also able to investigate the dynamics of hazard and survival with respect to changes in level of gene expression.

### Dynamics of survival and hazard

The temporal dynamics of hazard and survival curves for the combined model follow from the above determination of parameter values. In the following we work out the details of this time course, as well as the influence of the covariates, with particular attention to the gene expression variables and their interactions. In order to isolate the effects of one or two of the variables within the combined model we will hold all other variables at a fixed level, usually the median. We begin with the seventy gene signature 70G, both in relation to the other gene expression variables CSR and CERBB, and also in comparison to 70G as a single variable model.

**Figure 1:**
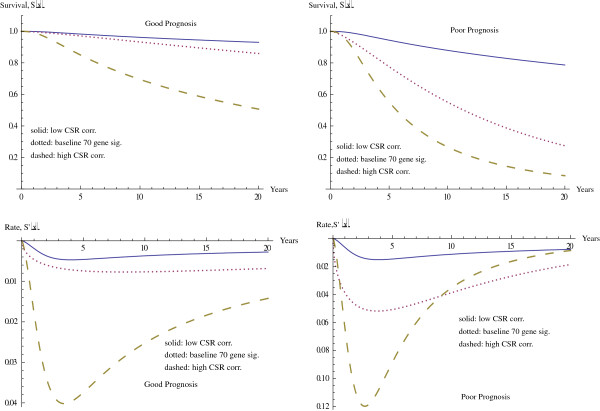
Survival function for varying CSR correlation and seventy gene signature.

We now analyze the interaction between the seventy gene signature and CSR correlation within our multivariable model, while holding our other variable of ErbB2+ correlation fixed at its median value. The graphs in Figure 1 show the interrelation between the seventy gene signature and the CSR correlation for survival functions and their derivatives. The axes on the left contain the curves for a seventy gene signature with a good prognosis, while the curves on the axes on the right have a seventy gene signature for a poor prognosis. The graphs on each set of axes represent a passage from the minimum CSR correlation at the top to the maximum CSR correlation at the bottom, while the curve in the middle represents the survival curve when only the seventy gene signature is considered. These are followed by the graphs of the rate of change of survival.

Notice that when the seventy gene signature has a poor prognosis, the effect of CSR correlation on survival is also magnified. We can determine the maximum rate of decrease in survival probability for each of the cases, and these are given in Table 3.

**Table 3:** Maximum rates of decrease in survival and increase in hazard with varying CSR

		**Time of min**	**Veloc. at min**
**Survival**			
Good prognosis (70G = 0)	CSR min	4.003	−0.004703
	CSR max	3.446	−0.04187
	Only 70 gene sig.	8.332	−0.007713
Poor prognosis (70G = 1)	CSR min	3.814	−0.01516
	CSR max	2.682	−0.1198
	Only 70 gene sig.	3.743	−0.05187
**Hazard**			
Good prognosis (70G = 0)	CSR min	2.187	0.006365
	CSR max	2.187	0.05976
	Only 70 gene sig.	5.107	0.009010
Poor prognosis (70G = 1)	CSR min	2.187	0.02111
	CSR max	2.187	0.1982
	Only 70 gene sig.	5.107	0.07695

We note that in the case of a poor prognosis for the seventy gene signature, the maximum rate of decrease in the survival function occurs sooner in all of the cases. Furthermore, this rate of change has a larger magnitude, indicating a larger rate of decrease in the survival function, when there is a poor prognosis. These graphs also compare the curve in the middle, where 70G is the only covariate with the curves on the outside. For these curves all four variables are included in the model, while the focus is on the variation in CSR correlation from the minimum value to the maximum value, with other variables at median level. Here the differences in shape also come about due to the variation in the values of α and β between these cases, a feature of the hypertabastic distribution allowing greater variability in the location and magnitude of the maximum rate of decrease for the survival functions.

**Figure 2:**
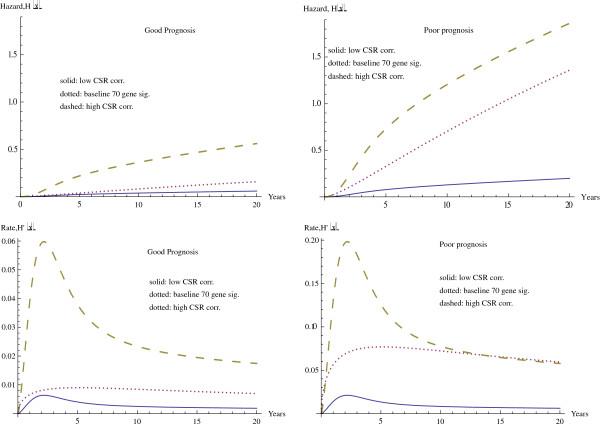
Hazard function for varying CSR correlation and seventy gene signature.

Figure 2 contains the hazard curves, together with their derivatives, for the same set of covariates. The significant difference appears again between graphs on the left, where the seventy gene signature shows a good prognosis and the graphs on the right, where it shows a poor prognosis. Again this shows the much larger effect of CSR correlation in the case of the poor prognosis. For instance at 20 years the difference in hazard values for minimum and maximum CSR is 0.5014 for a good prognosis, while this difference increases significantly to 1.663 for a poor prognosis in seventy gene signature. Table 3 shows the time and magnitude for the maximum rate of change of the hazard value.

For the two correlation variables (CSR and CERBB), an increased level of correlation is associated with a poor outcome, and both cases exhibit the same general profile of more invasiveness, more resistance to treatment, and shorter times until recurrence. In the following we compare the effect of the ErbB2+ correlation (CERBB) to the CSR correlation (CSR) treated above. We note that although there are some similarities, these biological processes measured by the two gene expression variables play different roles in tumor progression. The CSR correlation treated above deals with the role of fibroblasts in both wound healing and tumor progression in cancer and relates to the proposed wound-like phenotype that has been observed in a number of human cancers [10]. The CSR gene signature includes genes for cell motility, matrix remodeling, and angiogenesis, which correspond to increased risk of metastasis and the potential for a more invasive cancer. This signature gives a strong prediction of outcome in several cancer types. The role of ErbB2 in determining outcome has been established in numerous studies [29] and is independent of other prognostic factors. These protein tyrosine kinases in the HER (ErbB) signaling network play critical roles in cell signaling that regulate proliferation, migration, and survival [30]. Disruption of the signaling network of tyrosine kinases figures prominently in many known oncogenic mutations leading to neoplasms, including cases of breast carcinomas. HER2/neu has also been shown to disrupt the p53 tumor suppression pathway [31]. The action of this signaling network and its role in cancer progression continues to be studied in order to discover new therapies.

The different means of action between ErbB2 and CSR allows for overlap of both these variables in determination of probability of survival. The effect of ErbB2+ correlation (CERB) in the survival model follows approximately the same pattern as the CSR correlation (CSR) described above, although the magnitude is somewhat smaller, as described below. The hazards ratio and p-values for these two variables are comparable when considered individually, with hazard ratios of (45.489) and (30.036) for CSR correlation and ErbB2+ correlation, respectively, and p-values of (1.462 10^-9) and (2.990 10^-7), respectively. However, when considered with all the other variables in the model, these become hazard ratios of (14.305) and (4.766) for CSR correlation and ErbB2+ correlation, respectively, and p-values of 0.0001524 and 0.03208, respectively. The effect of the seventy gene signature on the ErbB2+ correlation will be comparable to the effect on the CSR correlation, as demonstrated above. Thus the ErbB2+ correlation will display the same pattern as the CSR correlation, with a somewhat smaller magnitude due to the difference in hazard ratios. In the following we will also investigate each of these correlations, CSR and ErbB2+, as continuous variables within our overall model. We will also consider the relation between these variables below, where an increase in correlation of one variable can be expected to amplify the effects of the other, as observed above for the seventy gene signature.

**Figure 3:**
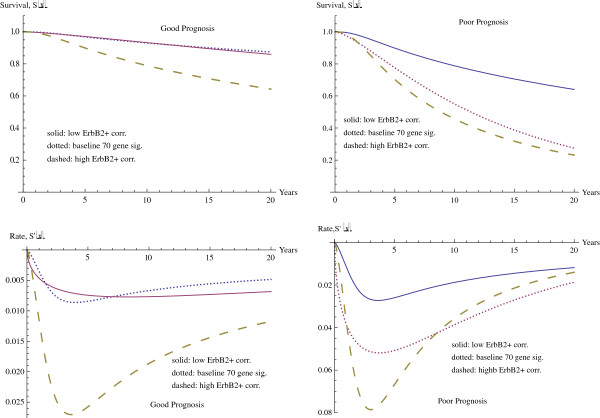
Survival function with varying ErbB2+ correlation.

The graphs in Figure 3 show the survival curves, together with the derivatives, with the case where only the seventy gene signature (solid curve in center) is considered compared with the four variable model for varying levels of ErbB2+ correlation. The curves on the outside represent the minimum level of ErbB2+ (dotted curve at top) and the maximum level of ErbB2+ correlation (dashed curve at bottom), with the good seventy gene signature in the axes on the left and the poor seventy gene signature in the axes on the right. The location and velocities of the minima for the rate of change of the survival curve are given in Table 4.

**Table 4:** Maximum rate of decrease of survival function with varying ErbB2+ correlation

		**Time**	**Velocity**
Good prognosis	Min ErbB2+	3.929	−0.008628
	Max ErbB2+	3.627	−0.02704
	O7 only	8.332	−0.007713
Poor prognosis	Min ErbB2+	3.624	−0.02722
	Max ErbB2+	3.024	−0.07859
	O7 only	3.743	−0.05187

The effect of the ErbB2+ correlation is comparable to that for CSR correlation observed above, although the magnitude is smaller. The difference in 20 year survival rates between the minimum and maximum ErbB2+ correlations are 0.2316 in the case of good seventy gene signature and 0.4097 in the case of poor seventy gene signature. These are just over half of the effect observed for the difference between minimum CSR correlation and maximum CSR correlation, which is 0.4235 for the good seventy gene signature and 0.7021 for the poor seventy gene signature.

In the remainder of the study we further describe interactions between our three gene expression variables, 70G, CSR, and CERBB, in determining the survival function. As the variables for CSR correlation and ErbB2+ correlation are continuous variables, we study the effect of variation of the level of correlation on the survival function. We first investigate separately the effects of each of these correlations, CSR and ErbB2+, in determining the probability of survival beyond ten years. Then, as a function of two variables we are able to investigate the combined effect of these two correlations on the probability of survival beyond ten years. We also use two variables to consider the effect of each of these individual variables in combination with time. In each case we analyze the survival function to explore quantitatively how change in the level of correlation will affect the prognosis and the probability of survival beyond a given time. It is also possible to determine at what time a given correlation will display its largest impact on survival. This analysis will further allow us to compare the influence of these two variables, CSR correlation and ErbB2+ correlation, and how they affect the survival and hazard curves, over time.

**Figure 4:**
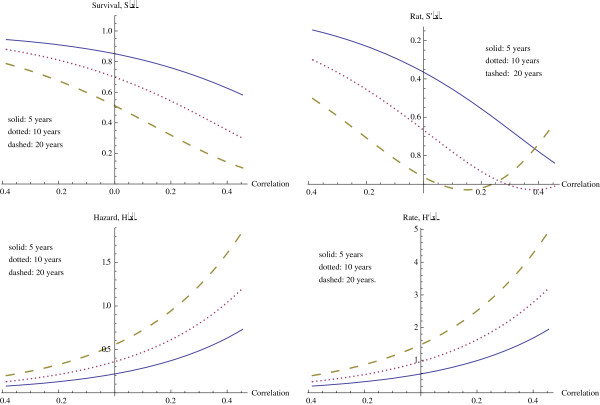
Survival and hazard at 5, 10, and years, as functions of CSR correlation.

We first investigate the role of CSR correlation (CSR) while holding the other variables at median level and assuming a poor prognosis in seventy gene signature (70G). We consider three fixed times, probability of survival past 5 years, past 10 years, and past 20 years. These survival curves, followed by their rates of change, are given in Figure 4. The horizontal axis for CSR correlation varies from the minimum CSR correlation to the maximum CSR correlation for the data set, and our interest is primarily in this range of values for CSR correlation.

As expected, survival drops off with increasing CSR correlation. The effect from the CSR correlation increases with time, as may also be expected. For survival beyond 5 years, the decrease in survival with increasing CSR correlation occurs at an increasing rate throughout the experimental range of CSR correlations, reaching a maximum rate of decrease of (−0.8387) at the maximum correlation. However at 10 and 20 years, the effect of CSR correlation in decreasing survival is even larger, with a maximum rate of decrease occurring at correlations within the experimental range. The specific values are given in Table 5. Clearly, as time increases the CSR correlation has a larger effect, with significant effects noticeable at much lower levels of correlation. Similarly, at the minimum values of correlation the effect of time is much less significant, and the survival rates are much higher.

**Table 5:** Maximum rate of decrease for survival function with CSR correlation vs. EbB2+ correlation

	**Time**	**Correlation**	**Velocity**	**Correlation**	**Velocity**
Effect of variation of CSR correlation	5 years	0.6855	−0.9788	Max	−0.8387
	10 years	0.3855	−0.9788	0.3855	−0.9788
	20 years	0.1498	−0.9788	0.1498	−0.9788
Effect of variation of ErbB2+ correlation	5 years	1.000	−0.5652	Max	−0.3868
	10 years	0.6063	−0.5744	Max	−0.5590
	20 years	0.2063	−0.5744	0.2063	−0.5744

Note: Max[CSR] = 0.455306 and Max[CERBB] = 0.451045 for this data set.

The hazard function continues increasing for both increasing time and increasing correlation, as we observe in the hazard graphs found in Figure 4.

**Figure 5:**
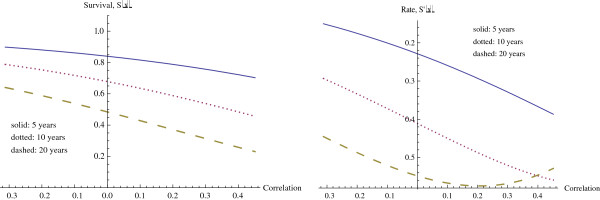
Survival at 5, 10, and 20 years, as functions of ErbB2+ correlation.

We now investigate how ErbB2+ correlation affects the probability of survival beyond times of 5, 10, and 20 years. The graphs representing these survival curves appear in Figure 5. The general effect is the same as that just observed with CSR correlation, but of a smaller magnitude. Along with a smaller overall magnitude of effect, the correlation must also reach higher levels in order to achieve its level of maximal effect. Table 5 also describes the maximum rate of decrease in these survival functions and the corresponding ErbB2+ correlations. In comparison with the CSR correlation, the rates of decrease of survival with respect to ErbB2+ correlation are considerably lower, with the maximum rate of decrease for the CERBB variable being approximately half that of the CSR variable, and requiring a higher level of correlation.

To further illustrate the quantitative difference for these two variables, we give Table 6 below, representing probability of survival beyond 10 years at several levels of CSR correlation and ErbB2+ correlation. For the CSR columns, the ErbB2+ correlation is held at its median, and likewise CSR correlation is fixed at its median level for the CSR column. The stronger influence of the CSR correlation on survival can be seen in the wider variation in the range of survival probabilities with CSR correlation.

**Table 6:** Probabilities of 5 year and 10 year survival with varying CSR and ErbB2+ correlations

**5 year survival:**					
**Correlation**	**CSR**	**ErbB2+**	**Correlation**	**CSR**	**ErbB2+**
−0.3	0.9299	0.8967	0.1	0.8101	0.8157
−0.2	0.9095	0.8803	0.2	0.7597	0.7881
−0.1	0.8836	0.8615	0.3	0.6987	0.7570
0	0.8509	0.8401	0.4	0.6263	0.7223
**10 year survival:**					
**Correlation**	**CSR**	**ErbB2+**	**Correlation**	**CSR**	**ErbB2+**
−0.3	0.8506	0.7844	0.1	0.625607	0.6354
−0.2	0.8097	0.7528	0.2	0.542263	0.5885
−0.1	0.7592	0.7177	0.3	0.44998	0.5380
0	0.6981	0.6784	0.4	0.352761	0.4845

**Figure 6:**
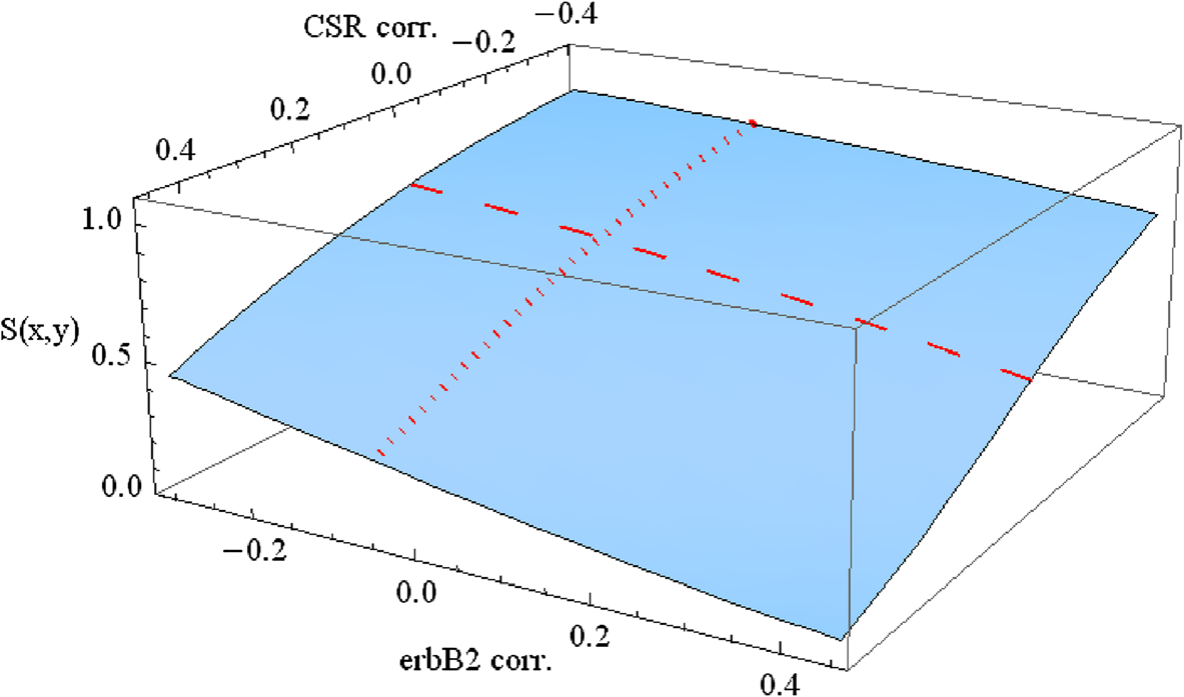
Survival beyond 10 years for CSR and ErbB2+ correlation.

We consider how the survival function depends on both of these continuous variables. Note that since Table 6 always fixes one of these variables at the median level, it will not show either the highest or lowest extremes. In order to study the dependence of survival on both CSR correlation and ErbB2+ correlation, it is necessary to consider the survival function S[x,y,t] as a function of the variables x(CSR), y (ErbB2), and time t. We can represent S[x,y,t_0_] as a three dimensional graph for any fixed value of t_0_. In Figure 6 we consider survival beyond 10 years, letting t_0_ = 10. The other variables are fixed at median age and a seventy gene signature representing a poor prognosis.

The dotted and dashed curves along the surface of this graph correspond to the 10 year (dotted) survival curves in Figures 4 and 5, respectively. These are the cases of varying CSR correlation (CSR) at the median level of ErbB2 correlation (CERBB) and of varying ErbB2 correlation (CERBB) at median CSR correlation (CSR), respectively. The values in Table 6 above correspond to the appropriate points along these curves. Inspection of the surface of the graph in Figure 6 shows clearly that a much wider range of interaction of these variables CSR and CERBB is possible beyond the points on the two curves.

**Figure 7:**
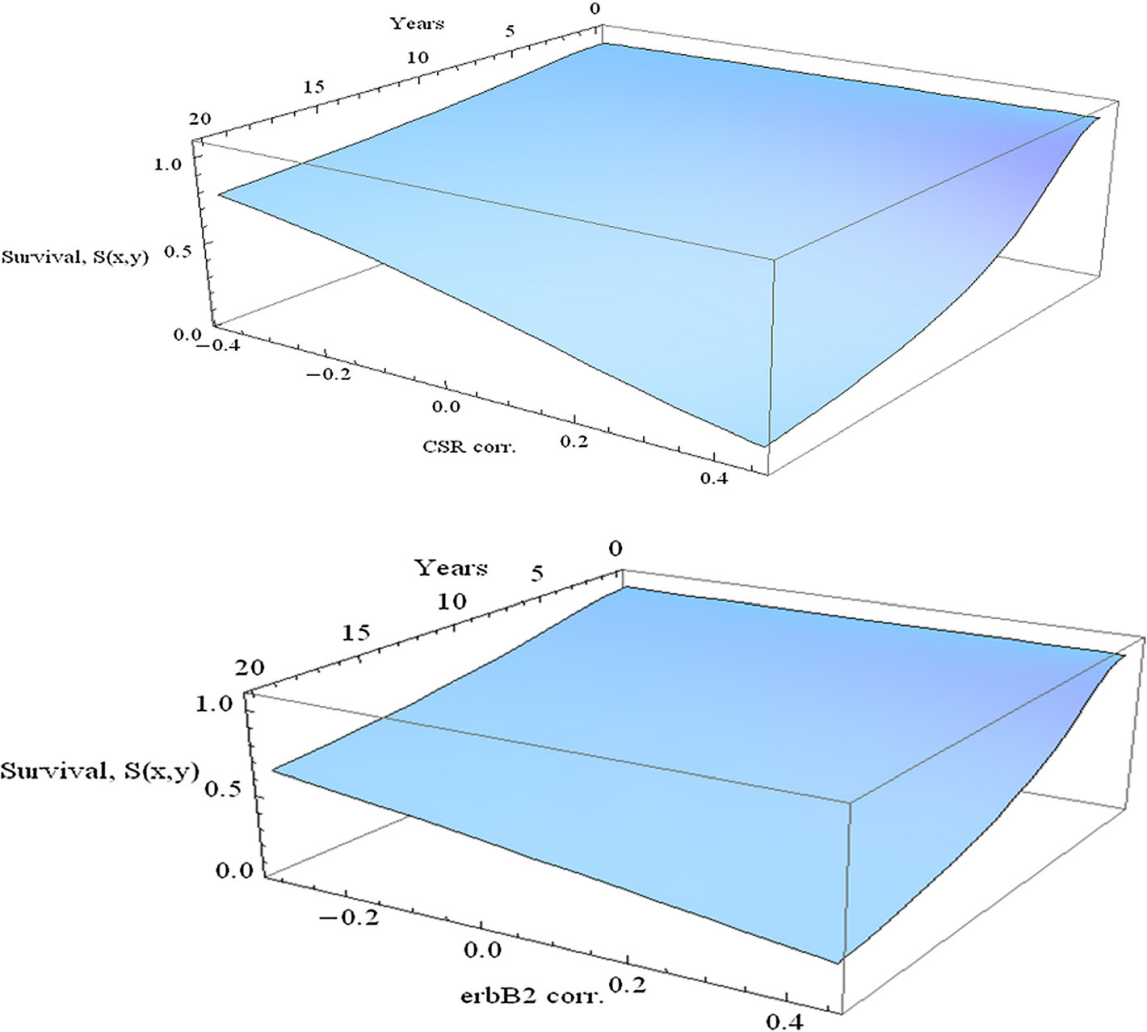
Survival as a function of time and correlation.

The graph in Figure 6 and the above computations describe the interaction of the two correlation variables for the fixed time of 10 years. In Figure 7 we explore how each of the variables CSR and CERBB interacts with time in predicting survival. In each of these three-dimensional graphs a poor prognosis is assumed from the seventy gene signature, while the other variables are held at the median level.

The comparative effects of CSR correlation and ErbB2+ correlation are obvious from these graphs. At each time change of CSR correlation has a much larger impact as compared to ErbB2+ correlation. Similarly, for each given level of correlation, the decrease of survival percentage with respect to time is much larger for CSR correlation.

Since the function (2) with the parameter values estimated by the model contains all of this information, it is possible to compute probabilities of survival to any time for any given combination of the variables. As a representative examples of the types of computations that can be made, in Table 7 we give probability of survival beyond 10 years, probability of survival beyond 20 years, and the conditional probability of survival beyond 20 years given survival to 10 years. The variables are at median level unless otherwise mentioned. Low levels of CSR or ErbB2+ correlation correspond to the tenth percentile, while high levels correspond to the ninetieth percentile.

**Table 7:** Explicit computation of survival probabilities for representative cases

		**10 years**	**20 years**	**20 years | 10 years**
	Good prognosis	0.8988	0.8193	0.9116
	Poor prognosis	0.7020	0.5164	0.7357
Good prognosis	Low CSR	0.9428	0.8958	0.9502
	High CSR	0.8114	0.6769	0.8342
	Low ErbB2+	0.9214	0.8583	0.9315
	High ErbB2+	0.8420	0.7253	0.8614
Poor prognosis	Low CSR	0.8225	0.6942	0.8440
	High CSR	0.5001	0.2742	0.5482
	Low ErbB2+	0.7624	0.6024	0.7903
	High ErbB2+	0.5654	0.3447	0.6097

In this four-variable model we observed how each of the three gene expression variables influenced the survival and hazard functions for breast cancer patients. For the two continuous gene expression variables, CSR correlation and ErbB2+ correlation, we analyze the effect of changes in levels of gene expression. We were able to assess the combined effect of these variables, or we could look at them separately and compare their effects, such as the above comparison of effects of change in CSR correlation and ErbB2+ correlation. The feature of the hypertabastic survival model of producing explicit hazard and survival functions allowed us to analyze these dynamics. Additionally we are able to compute explicit survival probabilities for any given patient profile. In concluding this survival analysis using several clinical and gene expression variables, we mention our recent work [3], in which we investigate the role of metastasis in survival analysis and its interactions with the other covariates.

## Conclusions

The new model presented in this article combines several features not included in previous models in survival analysis of breast cancer patients. Through use of the hypertabastic survival model, a parametric model we attain a better fitting model. It furthermore offers explicitly defined hazard and survival functions for use as tools in analysis. As demonstrated in this article, these functions can be used for computation of probabilities, such as those given in the tables above. Furthermore, analysis of the time course of these functions allows scientists to study the time course of the progression of hazard and the decline in survival for these patients. The influence of the variables, collectively or individually, can also be investigated in their role in determining this time course. This analysis illustrates the value of parametric models in survival analysis in cases where a suitable distribution can be found to be close enough to the underlying distribution of the data. We recommend consideration of the hypertabastic distribution as it is shown in [3] and in the current paper to have a good fit to breast cancer survival data. Furthermore simulations [2] have shown it to be robust with respect to departure from distribution. The feature of the hypertabastic distribution in adjusting its shape for a more accurate representation of the time course of the hazard and survival functions. In the context of the current work of scientists in developing gene expression variables for clinical use, these novel features of this model become even more significant.

The novel feature of the current model of investigating collective behavior of distinct gene expression variables offers an important new direction of research. The three gene expression variables included in this model originate from three distinct types of gene expression signatures: one signature representing early distant metastasis, one representing the relation of the wound healing microenvironment to that of tumor progression, and the third representing classification of breast cancer tumors into molecular subtype. Furthermore the model gives a means to determine the relative contribution of each variable, quantitatively, in determining survival and hazard. For the two continuous gene expression variables we were also able to investigate the rate of change of hazard and survival with respect to change in the level of gene expression.

By consideration of a wider range of gene expression variables together with clinical variables, this model has moved beyond previous models toward a quantitative assessment of hazard and survival involving all relevant information. These results show the potential to use multiple gene expression signatures to a combined greater effect when the signatures represent different aspects of the cancer biology. We note however that the current model has limitations in its representation of potential interactions between the various gene expression signatures. We feel this issue of interactions among gene expression variables, as well as other variables, is a critical issue for current research. We propose further investigations in this direction, as well as development of new and more refined models designed for this purpose. Certainly the new generation of gene signatures being developed for clinical use [17,18] should also be explored for their potential interactions and combined effects. As an extension of this work, we have explored the effect of an additional variable representing metastasis in a recent paper [3], particularly in relation to the other variables in the model. We also propose to make a similar analysis after dividing the breast cancer cases into several different classes, such as estrogen receptor positive versus estrogen receptor negative cancers, or for the molecular subtypes based on the correlation variables CNorm, CERBB, CLumA, CLumB, and CBas. Another important direction for future research will be identification and analysis of variables that either cause the metastasis of tumors or that accelerate this process.

## Abbreviations

ErbB2: v-erb-b2 erythroblastic leukemia viral oncogene homolog 2; HER2: Human epidermal growth factor receptor 2; CSR: Core Serum Response; AIC: Akikake Information Criterion; ER: Estrogen Receptor.

## Competing interests

The authors declare they have no competing interests.

## Authors’ contributions

The work presented in this paper was carried out in collaboration among all authors. M.A.T and W.M.E. applied the hypertabastic proportional hazards model for the breast cancer data, analyzed and interpreted the data, and wrote the paper. N.N and K.P.S. participated in the interpretation and analysis of the data and gave technical assistance. H.L. assisted with running the SAS aspects of the program for the hypertabastic proportional hazards model, as well the log-logistic, Weibull, and Cox regression cases. H.L. also participated in discussion of the results. All authors read and approved the final manuscript.

## Pre-publication history

The pre-publication history for this paper can be accessed here:

http://www.biomedcentral.com/1755-8794/5/63/prepub

## Supplementary Material

Additional file 1 Data cancer. Click here for file
