# Supplementary material for: Clinical and multiple gene expression variables in survival analysis of breast cancer: Analysis with the hypertabastic survival model
Source: BMC Med Genomics. 2012 Dec 14;5:63. doi: 10.1186/1755-8794-5-63 (PMC3548720; doi:10.1186/1755-8794-5-63)
Supplement: Additional file 1 — Data cancer. [file 1755-8794-5-63-S1.docx]

**data** cancer;

infile 'C:\Documents and Settings\TENSL\My Documents\ Brestcancer_Data_2.csv' Delimiter=',' DSD Missover;

input age geneo $ CSR Erbb status time;

**data** cancer; set cancer;

/*proc print; 295 obs;*/

title1 'Hypertabastic proportiional hazard model-Log time'; /*fit model 1*/

**data** cancer; set cancer;

if geneo='Poor' then gene7=**1**;

if geneo='Good' then gene7=**0**;

title1 'Hypertabastic proportiional hazard model-Log time';

**Proc** **nlp** data=cancer tech=quanew cov=**2** vardef=n pcov phes maxiter=**250**;

max logf;

parms a=**0.01**, b=**0.1**, c=**0.01**, f=**0.1**, i=**0.1**, k=**0.1**;

y=status;

in1=time**b;

in2=tanh(in1);

in3=a-a*(in1)/in2;

in4=in3/b;

in5=tanh(in4);

in6=exp(c*age+f*Erbb+i*gene7+k*CSR); /*covariates*/

/*likelihood equation*/

s= log(**1**/cosh(a*(**1**-in1* **1**/tanh(in1))/b)) * in6 + y*log((a*(-**1***in1 * **1**/tanh(in1)+

time**(**2***b)* **1**/sinh(in1)****2**) * in5) * in6);

logf=s;

**run**;
